# Predicting the Antiretroviral Medication Adherence and CD4 Measure in Patients with HIV/AIDS Based on the Post Traumatic Stress Disorder and Depression

**Published:** 2019-01

**Authors:** Zeinab EBRAHIMZADEH, Mohammad Ali GOODARZI, Hassan JOULAEI

**Affiliations:** 1. Department of Clinical Psychology, School of Education and Psychology, Shiraz University, Shiraz, Iran; 2. Shiraz HIV/AIDS Research Center, Shiraz University of Medical Sciences, Shiraz, Iran

**Keywords:** Antiretroviral medication adherence, CD4 cells, Depression, Post traumatic stress disorder

## Abstract

**Background:**

Antiretroviral therapy has significantly reduced the prevalence of diseases and mortality rate caused by HIV; therefore, recognition of the factors affecting the antiretroviral therapy is of great importance. We aimed to investigate the relationship between antiretroviral medication adherence and CD4 with posttraumatic stress disorder (PTSD) and depression in patients with HIV.

**Methods:**

This was a descriptive, cross-sectional, quantitative, and correlational study. The statistical population included all of the patients with HIV in Shiraz, Fars Province, southwest of Iran in 2013, of whom 220 were selected from the Behavioral Diseases Consultation Center using the convenience sampling method. The measures included Mississippi Post Traumatic Stress Disorder Questionnaire, Beck-II Depression, and ACTG Adherence (ACTG). The results were analyzed using the Pearson correlation method and stepwise hierarchical multivariate regression.

**Results:**

Regression analysis showed that of two mediating variables (age & educational level), only age could predict 5% (*P*<0.001) and of two predictive variables (depression & PTSD) only PTSD could predict 53% (*P*<0.001) of medication adherence's variance. Moreover, of two mediating variables (age & disease duration), only age could predict 3% (*P*<0.004) and of two predictive variables (depression & PTSD) only PTSD could predict 4% (*P*<0.001) of CD4 variance.

**Conclusion:**

The posttraumatic stress disorder symptoms could predict the medication non-adherence and lower CD4 levels.

## Introduction

The HIV/AIDS epidemic has been widely acknowledged as one of the world's most serious health crises of this century (1). This disease is caused by immune deficiency, which leads to immune weakness via destruction of the immune cells, notably the CD4 cells (2). Until Mar of 2016, 31950 persons with HIV have been recognized in Iran, of whom 84% are men and 16% are women (3).

Further studies developed new strategies for antiretroviral medication (4). The advent of highly active antiretroviral therapy (ART) prominently decreased AIDS-related mortality such that approximately 30 million people currently live with HIV around the world (5, 6). High levels of adherence to the medication are necessary for successful medication (7). However, considering the availability of ART, people living with HIV (PLWH) are not uniformly responsive to treatment (8) and there is nearly 50% to 80% of non-adherence to the medication (9–11) and poor adherence is a primary factor in suboptimal treatment response (12). The reasons for non-adherence are various (13); side effects of the antiretroviral medication such as nausea or anemia and a number of psychological factors play an important role in the impaired adherence to the antiretroviral therapy (14). These include the patient's low self-efficacy, psychological distress, depression, trauma, amnesia, substance use disorders, and low social support (15–17). Moreover, distress psychological symptoms are associated with disease progress through adherence reduction (18).

Patients with HIV often experience depression and posttraumatic stress disorder (PTSD) simultaneously (19, 20). Prevalence of the depression in these patients has been reported as 40%–50% (21). The incidence of stress-related disorders, such as PTSD, is elevated among people living with HIV as compared to those living without the virus (22). There is a relationship between trauma symptoms, as seems in PTSD (avoidance of memories of the traumatic event, recurring flashbacks, hyperarousal) and experiencing various medical conditions (23, 24) like positive HIV (25) that Trauma symptoms in patients with HIV are usually linked to deterioration in immune function through reducing CD4 cell counts and increasing the level of physical HIV symptoms (26) and poor medication adherence (27). More symptoms of depression in HIV patients are associated with lower adherence to the medication diet (28–32) and low rate of the CD4. The patients with more depression symptoms, have the low rate of CD4 cells and probably have the low rate of adherence to the diet compared to those patients with more PTSD symptoms and patients with more symptoms in both disorders (depression and PTSD) (28). Additionally, increase in PTSD symptoms is associated with the reduction in medication adherence (31–36). Depression with no use of the anti-depression drugs is linked to the medication adherence reduction (37, 38). In Iran, psychiatric disorders, psychological variables or quality of life were investigated in patients with HIV/AIDS or attitude and knowledge about this illness in various groups (39–44) and the quality of life and mental health in people affected by HIV are lower than healthy people and they suffered from substance-related disorders, mood disorders and anxiety disorders (42–44). However despite importance of antiretroviral medication adherence, furthermore, it's effect on HIV/AIDS patients' well-being or healthy, this issue has not been investigated in Iran.

Therefore, considering how high prevalence of psychiatric disorders in HIV patients in Iran (42–44) and also importance of the antiretroviral medication adherence and CD4 rate, we investigated the relationship between medication adherence and CD4 rate with two mental disorders (depression and PTSD) in these patients.

## Materials and Methods

This study was implemented in Shiraz, Fars Province, southwest of Iran in 2013. Study population was total registered HIV^+^ patients who referred from all around Fars Province to Shiraz voluntary counseling and testing center.

### Sampling Method

By using convenience sampling method, 220 patients (129 males & 91 females) were selected and examined. These patients who received the antiretroviral medication, did not suffer from any other physical disease, were not affected by severe psychological disorder or psychotic disorders such as Schizophrenia or Mania-Depression included in this study. Furthermore, they had the minimum level of reading and writing skill to complete the questionnaires based on their own reports.

### Data Collection Method

In order to collect the demographic data, drug adherence and PTSD situation of the patients, investigators applied different questionnaires. Moreover, CD4 levels were obtained using the medical files of the patients. Each questionnaire is explained briefly as follow;

### Demographic Characteristics questionnaire

In order to evaluate the demographic characteristics such as age, gender, employment, marital status, education, income, disease duration, and duration of the treatment, a researcher-developed questionnaire was used.

### Medication Adherence Questionnaire of AIDS Clinical Test Group (ACTG)

This questionnaire was used for measuring the antiretroviral medication adherence (45). Using Cronbach's Alpha Method, the questionnaire's reliability was calculated as 0.79. Furthermore, its criterion (simultaneous) validity was measured by counting the pills, which is another method for measuring the adherence rate. Medication adherence index in pill counting was calculated using patient's medical files and based on following formula:

Medication adherence=number of pills actually used by the patient/number of pills that the patient should have used × 100˙

Pearson's correlation coefficient was 0.82 (*P*<0.01) between the score of medication adherence questionnaire and score of medication adherence based on pill counting.

### Mississippi (Echelle) PTSD measure:

This questionnaire has been developed. The items have been manipulated based on the Iranian culture in 2004 and included 39 items scored from 1 to 5 according to the Likert scale. The total score range was from 39 to 195. Cronbach's alpha coefficient was reported as 0.92 and test-retest reliability over week was 0.91. Furthermore, concurrent validity of the questionnaire was measured via its correlation with PTSD symptom list, that the resulting coefficient was 0.82 (46).

### Beck Depression Inventory (BDI-II):

This Inventory with 21 items was introduced in 1961. Each item rated on a 3 point Likert scale. Total scores range was from zero to 63 (47). In Iran, this Inventory was examined in a sample including 354 people. The reliability coefficient at one week was reported as 0.93 and its convergent validity With Hamilton's revised of psychiatric classification for depression was reported as 0.71 (48).

### Statistical Method

This was a descriptive, cross-sectional, and quantitative (correlational) study. Results were analyzed using the SPSS software (Chicago, IL, USA), descriptive and inferential statistics, correlation method and stepwise hierarchical multiple regression. In order to examine the reliability of the medication adherence questionnaire, Cronbach's alpha and in order to examine the concurrent validity with the tablet count method, correlation method was used.

### Ethical approval

The study proposal was approved by the Research Ethics Board of Faculty of Educational Sciences of Shiraz University. Also, written informed consent was received from the participants prior to their participation in this study.

## Results

Two hundred twenty subjects participated in this study (58/6% men, 41/4% women) of which 112 (50.9%) showed symptoms of depression (based on the Beck Depression Inventory cut off point of 18) and 42 (19.1%) showed the PTSD symptoms (based on the Echelle PTSD Questionnaire cut-off point of 107). According to a large number of the studies consider 95% or more use of antiretroviral medicines as adequate adherence (49, 50), 78 (35.4%) were identified as adherent and 142 (64.5%) as non-adherent to the treatment (Table 1).

**Table 1:** Descriptive statistics for the study variables

***Characteristic***	***Mean***	***Standard Deviation***
Age(yr)	38	7.67
Education (year)	6.08	1.23
Income (Rial)	3067700	2405400
Disease Duration (month)	70.75	46.7
Treatment Duration (month)	30.1	29.2
CD4 Rate	255.3	154.3
Medication Adherence	14.91	4.07
Depression	20.78	13.84
PTSD	82/80	25/99

Correlation results showed that there was a significant negative relationship between age and medication adherence (r=−0.20, *P*<0.01), age and CD4 rate (r=−0.19, *P*<0.01), disease duration and CD4 rate (r=−0.14, *P*<0.05) as well as a significant positive correlation between medication adherence and educational level (r=+0.14, *P*<0.05) and CD4 rate and medication adherence (r=+0.20, *P*<0.01) (Table 2).

**Table 2:** Correlation matrix between demographic characteristics and the variables

***Scales***	***Age***	***Educational Level***	***Income***	***Disease Duration***	***Treatment Duration***	***CD4 Rate***	***Medication Adherence***	***Depression***	***PTSD***
Age(yr)	1								
Educational	−0.12 ^*^	1							
Level									
Income	0.05	0.28 ^**^	1						
Disease	0.27 ^**^	0.03	−0.06	1					
Duration									
Treatment	0.24 ^**^	−0.13 ^**^	−0.11	0.41 ^**^	1				
Duration									
CD4 Rate	−0.19 ^**^	−0.02	−0.02	−0.14 ^*^	0	1			
Medication	-	0.14 ^*^	0.10	−0.01	−0.08	0.20 ^**^	1		
Adherence	0.20 ^**^								
Depression	0.18 ^**^	−0.20 ^**^	−0.23 ^**^	0.09	0.10	−0.19 ^*^	−0.61 ^**^	1	
PTSD	0.19 ^**^	−0.14 ^*^	−0.20 ^**^	0.13	0.11	−0.19 ^*^	−0.76 ^**^	0.82 ^**^	1

** *P* < 0.01,

* *P* <0.05

However, there was a significant relationship between the age/educational level and antiretroviral medication adherence; so age and educational level were considered as the controlling (mediating) variables and a stepwise hierarchical multiple regression were used in order to explore the predictive value of variables. The effect of the first stage variables (age and educational level) was significant only for age (*P*<0.001). In the second stage, by adding PTSD and depression, prediction ability increased nearly 53%. Of these two variables, only PTSD significantly predicted medication adherence. Totally, age and PTSD explained about 58% of medication adherence's variance rate (*P*<0.001). Similarly, in order to predict the CD4 rate based on PTSD and depression severity, a further stepwise hierarchical multiple regression analysis was used. Using the hierarchical regression for predicting the CD4 rate, the effect of variables in the first stage was significant only for age (p<0.004). In the second stage, by adding PTSD and depression, prediction ability increased almost 4%. Of these two variables, only PTSD could predict CD4 rate significantly. Totally, age and PTSD variables explained about 7% of CD4 variance rate (*P*<0.001) (Table 3).

**Table 3:** Results of Multiple Regression Correlation

***Dependent variable***	***Regression*** ***progress steps***	***Entered variables***	***ß coefficient***	***t-test***		***f-test***		
				***t-coefficient***	***Significance***	***Adjusted R-square***	***f-coefficient***	***Significance***
Medication Adherence	First Step	Age	−0.19	−2.91	0.004	0.05	6.80	0.001
	Second Step	Age	−0.06	−1.36	0.004	0.58	103.32	0.001
		PTSD	−0.74	−16.70	0.001			
CD4 Rate	First Step	Age	−0.16	−2.43	0.004	0.03	5.26	0.004
	Second Step	Age	−0.16	−2.46	0.004	0.07	7.50	0.001
		PTSD	−0.19	−2.91	0.004			

## Discussion

The results indicated a significant negative relationship between age and medication adherence. In previous studies, no significant relation was observed between age and medication adherence (30, 33, 51–53) or medication adherence was higher in older patients (28, 54). Cognitive impairment in elderly patients might explain this negative relationship (45); for example, memory problems caused they forget to take their medication. This may be related to low educational levels in elderly people as indicated in our correlational analysis (r = −0.12, *P*<0.05). A negative relationship was observed between age and CD4 rate on one hand, and between disease duration and CD4 rate, on the other hand. By passing the time and increasing age, obviously the disease progresses and CD4 rate is reduced in the patients. There was a significant positive relationship between medication adherence and educational level, too. Patients with low literacy might more probably show non-adherence to diets (52). People with low educational levels had no enough information about HIV disease and notably the antiretroviral treatment. This might lead to ignoring antiretroviral medication diets for controlling the disease. There was no significant relationship between patient's income and medication adherence/CD4 rate. These results were consistent with some studies (28, 33, 50–53). However, in some studies, there was a significant relationship between personal income and medication adherence rate (30). Since free antiretroviral drugs were given to the patients, this result was expected. In addition, disease duration and treatment duration had no significant correlation with medication adherence. In comparison to patients who were medication adherent or who showed intentional medication non-adherence, patients who showed unintentional medication non- adherence, had longer duration of disease and treatment (54). In primary stages of the treatment process, patients might ignore the treatment and diet because of the disease non-acceptance; however, on the other hand, after a long time of the disease progression and treatment, these patients felt tired and might even conclude that treatment is not effective.

Only PTSD variable could significantly predict the medication adherence and CD4 rate. In previous studies, depression played a role in predicting lower medication adherence better than PTSD (28, 33). These research findings were consistent with a number of previous studies that revealed increasing PTSD symptoms was associated with reduction in medication adherence (32, 34–36). Depression could predict the antiretroviral medication adherence rate (29, 30, 37, 38). There is a high overlap percentage of the symptoms of these two disorders. In fact, most of the depression symptoms (sleep problems, feeling of guilt, continuous thinking about the past, tendency to suicide and crying, high excitation, lack of pleasure feeling and lack of concentration or decision-making power) are observed in patients with PTSD, too. In PTSD, patient experiences high rate of distress and mental obsession (e.g., continuous thinking about the event caused HIV virus) and this might increase ignorance rate of taking drugs on time. Moreover, in patients with PTSD, there are physiological symptoms, which are similar to the side effects of antiretroviral drugs (such as palpitations, transpiration, gastrointestinal problems, and feeling faint) and this fact might increase the ignorance of taking drugs. In other words, the patient mistakenly thought that these symptoms were the side effects of the drugs, while they were related to the patient's PTSD. Probably PTSD symptoms facilitated the progress of HIV disease by affecting patient's immune system and finally led to decrease of CD4 rate.

Our study had some limitations. We could not directly state whether PTSD symptoms causes low adherence or decrease CD4 rate because the cross-sectional design of this study was inherently limited in understanding causal processes and it can be assessed in future studies.

## Conclusion

The posttraumatic stress disorder symptoms could predict the medication non-adherence and lower CD4 levels.

## Ethical considerations

Ethical issues (Including plagiarism, informed consent, misconduct, data fabrication and/or falsification, double publication and/or submission, redundancy, etc.) have been completely observed by the authors.
